# ﻿*Castanopsiscorallocarpus* (Fagaceae), a new species from Royal Belum (Perak) in Peninsular Malaysia

**DOI:** 10.3897/phytokeys.219.95991

**Published:** 2023-01-18

**Authors:** Wei Harn Tan, Lisa Ong, Joeri Sergej Strijk

**Affiliations:** 1 Southeast Asia Biodiversity Research Institute, Chinese Academy of Sciences & Center for Integrative Conservation, Xishuangbanna Tropical Botanical Garden, Chinese Academy of Sciences, Subang Jaya, Mengla, Yunnan 666303, China Universiti Brunei Darussalam Bandar Seri Begawan Brunei; 2 School of Environmental and Geographical Sciences, The University of Nottingham Malaysia, Semenyih, Selangor, Malaysia Southeast Asia Biodiversity Research Institute, Chinese Academy of Sciences & Center for Integrative Conservation, Xishuangbanna Tropical Botanical Garden, Chinese Academy of Sciences Yunnan China; 3 Institute for Biodiversity and Environmental Research, Universiti Brunei Darussalam, Jalan Tungku Link, BE1410, Brunei Darussalam The University of Nottingham Malaysia Semenyih Malaysia; 4 Faculty of Science, Universiti Brunei Darussalam, Jalan Tungku Link, BE1410, Brunei Darussalam Southeast Asia Biodiversity Research Institute, Chinese Academy of Sciences & Center for Integrative Conservation, Xishuangbanna Tropical Botanical Garden, Chinese Academy of Sciences, Mengla Yunnan China; 5 Alliance for Conservation Tree Genomics, Pha Tad Ke Botanical Garden, 06000 Luang Prabang, Laos Alliance for Conservation Tree Genomics, Pha Tad Ke Botanical Garden Luang Prabang Laos

**Keywords:** *Berangan*, chinquapins, flora of Peninsular Malaysia, hill dipterocarp forest, Malayan chestnut

## Abstract

A new species from the Fagaceae family, *Castanopsiscorallocarpus* Tan & Strijk, is described from Royal Belum State Park in Peninsular Malaysia. Here, we provide technical illustrations, colour images and a description of its conservation status and the collecting locality, in addition to a comparative analysis with other species in the region. The solitary nut of *C.corallocarpus* has a morphologically unique cupule, lined with rows of thick coral-like spines not seen in other *Castanopsis* species.

## ﻿Introduction

*Castanopsis* (D.Don) Spach, is the third largest genus in Fagaceae, comprising ca. 134 species (Phengklai 2008; Li et al. 2015; Strijk 2022). Apart from limestone formations, *Castanopsis* species can be found in various habitats, ranging from lowland rainforest to montane forest and even harsh environments, like acidic heaths and peat swamps (Soepadmo 1972). Many seem to have narrow ecological or habitat preferences though, with species often found on ridges or crests and along margins and riverbanks. All *Castanopsis* species are medium to large trees and, like other Fagaceae species, are co-dominant in the closed canopy layer (Soepadmo 1972). The wide geographical distribution of *Castanopsis* is restricted to (sub-)tropical Asia, ranging from north-eastern India (Nepal, Bhutan and Assam), parts of eastern Asia (southern China, Korea and Japan) and southeast Asia (Indochina and Malesia). The genus has two major biodiversity hotspots, namely Indochina and Malesia (Soepadmo 1972). New taxonomic discoveries of *Castanopsis* are skewed towards Indochina with eight new species having been described in the last few decades (Phengklai 2004; Chen et al. 2010; Chen et al. 2011; Vuong and Xie 2014; Hoang et al. 2018; Mitra et al. 2019). Interestingly, no new species of *Castanopsis* has been described in Malesia over the last two decades.

The dipterocarp-dominated tropical rainforests of Peninsular Malaysia are part of the megadiverse Sundaland forest range (Myers et al. 2000). It is estimated that Peninsular Malaysia has at least 9,030 vascular plant taxa comprising 248 families and 1,651 genera (Yong et al. 2021). The Fagaceae family is an important component of the rainforest in Peninsular Malaysia, with a total of 72 species comprising four genera (i.e. *Castanopsis*, *Lithocarpus*, *Quercus* and *Trigonobalanus*). Mast fruiting phenomena in the Sundaland occur on an irregular suprannual scale (2–10 years) for many plant families, resulting in long periods of crop scarcity (Medway 1972). Unlike most mast-fruiting families, tropical Fagaceae communities fruit annually at unsynchronised times throughout the year, providing an important food source for many animals during periods of low fruit availability (Kaul et al. 1986). Species in the genus are known as ‘*Chinquapin*’ (not to be confused with two species in the North American genus of *Chrysolepis* (Fagaceae), which are also often referred to as such). Locally, the genus is known as *berangan* in Malay or the Malayan chestnut (Corner 1988). There are 20 described *Castanopsis* species in Peninsular Malaysia and, of these, five species are endemic to Malaysia (Cockburn 1972; Soepadmo 1972; Strijk 2022).

Royal Belum State Park is in the State of Perak, in the north of Peninsular Malaysia. The rainforest of Belum consists of undisturbed and pristine lowland dipterocarp, hill dipterocarp and lower montane forests. Belum is part of the larger Belum-Temenggor Forest Complex (BTFC) which has an area of ca. 3,500 km^2^. The BTFC is also part of a larger forest complex, as it shares a border with two adjacent protected areas in Thailand (Hala-Bala Wildlife Sanctuary and Bang La National Park) and forms part of the Central Forest Spine of Peninsular Malaysia. A product of the geological merger between the supercontinents of southern Gondwanaland and northern Laurasia, the landscape of BTFC is estimated to be more than 130 million years old, which is older than the Amazon and Congo Basins (Malaysian Nature Society 2005). Given its unique position within the Peninsula, the rich floristic composition of BTFC is a mixture of Thai-Burmese and Malayan flora with approximately 3000 species of flowering plants recorded, many of which are endemic to northern Peninsular Malaysia (Malaysian Nature Society 2005).

During a field expedition in Royal Belum State Park, Malaysia in July 2018, we came across a specimen with a large single nut and a unique burr that does not match any described taxa of *Castanopsis*. After examining the relevant literature on Malesian Fagaceae, we report this specimen as a new species, placed within the genus *Castanopsis*.

## ﻿Taxonomy

### ﻿ Castanopsis corallocarpus

Taxon classificationPlantaeFagalesFagaceae

W.H.Tan & Strijk sp. nov.

F867F3B7-2547-5411-A4BF-96E2307C8855

urn:lsid:ipni.org:names:77312007-1

Figs 1 , 2

#### Type material.

***Holotype*.** Malaysia, Sungai Tiang, Royal Belum State Park, Hulu Perak District, Perak State, elevation 417 m, 11 January 2022, *W.H. Tan TWH002*, Holotype: KEP; Isotypes: IBER [IBER00000000030; IBER00000000031]. ***Paratype*.** Malaysia, Sungai Papan, Royal Belum State Park, Hulu Perak District, Perak State, elevation 290 m, *W.H. Tan TWH003*, Paratype: KEP; IBER [IBER00000000032, IBER00000000033]. Due to the small number of individuals and precarious conservation status, detailed locality information is not released here, but can be requested from the authors.

**Figure 1.**
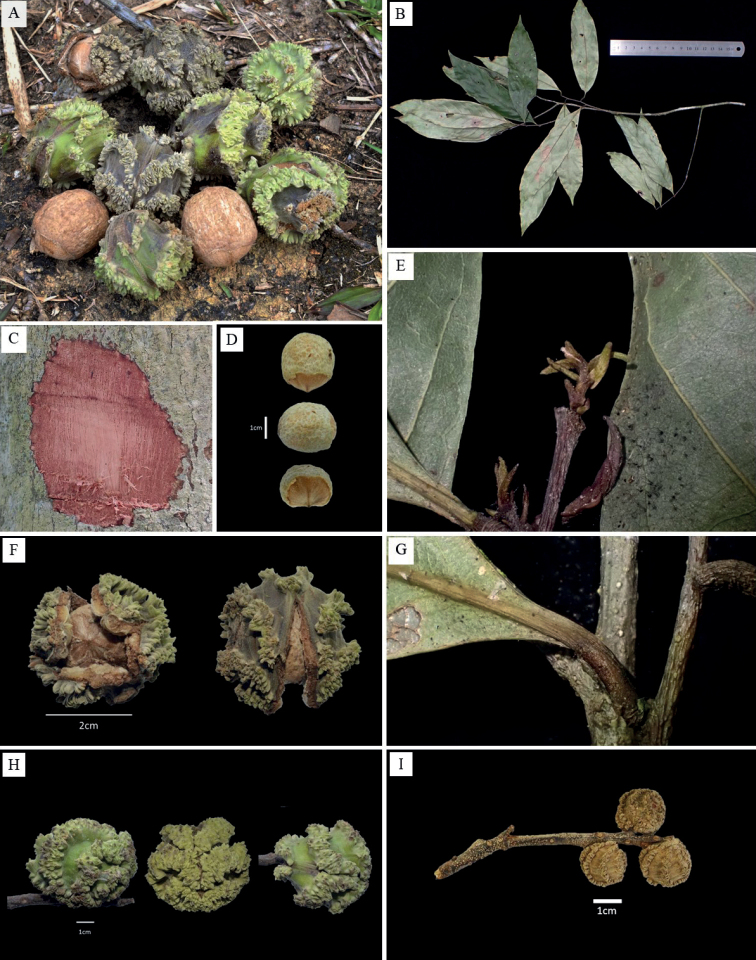
*Castanopsiscorallocarpus* W.H.Tan & Strijk, sp. nov. *W.H.Tan TWH002* (KEP) **A** fresh fruit from field collection **B** leaves and twigs from field collection **C** bark and sapwood **D** fresh nut – top view, side view, front view **E** young emerging leaf **F** mature fruit with splitting valves on the cupule – front view, top view **G** petiole **H** fresh mature – side view, front view, top view **I** young infructescence spike. All pictures by W.H.Tan and J.S.Strijk.

#### Diagnosis.

*Castanopsiscorallocarpus* is a medium-sized tree. It differs from similar species by its fruits which carry unique rows of basally reinforced, blunt coral-like spines on the cupule exterior, combined with singular rounded rectangular nuts which are slightly asymmetric. Currently, the species has been found in two localities, both in Royal Belum State Park and each consisting of one individual. Several additional individuals resembling *C.corallocarpus* were reported by our field staff in Temenggor Forest Reserve, Perak, but this is awaiting confirmation. Pending further discoveries, the species appears to be locally restricted to low-mid elevation forests of the BTFC.

**Figure 2.**
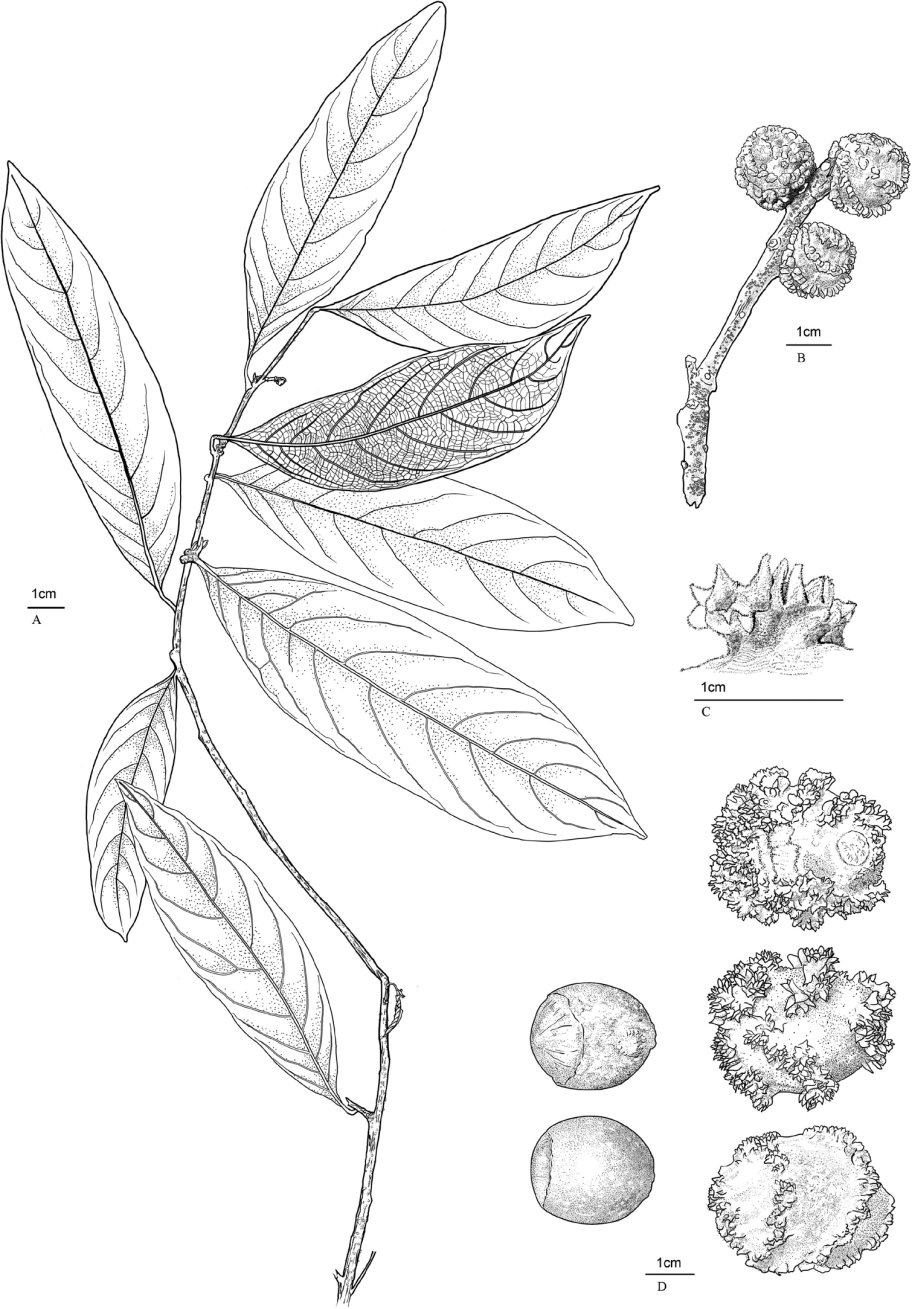
*Castanopsiscorallocarpus* W.H.Tan & Strijk, sp. nov. *W.H.Tan TWH002* (KEP) **A** habit **B** young infructescence spike **C** close-up of coral-like spine **D** mature nuts-bottom, top view and fruits-bottom, top and side view. Illustration by L. Ong.

#### Description.

Medium size tree, approximately 16–20 m tall, no buttresses. ***Bark*** lenticellate grey. Sapwood reddish-brown to pink. ***Branches*** glabrous, densely lenticellate, dark-brown to grey-white. ***Leaves*** simple, thin-coriaceous, papery when dried, lamina elliptic, up to (11)13–17(19) × (2)3.5–5(6) cm. Leaf apex acuminate; base cuneate, occasionally somewhat asymmetric. Margin entire. Both surfaces glabrous. Leaves dark green above and glaucous below. New shoots exhibit flushing. ***Venation*** pinnately veined, secondary venation eucamptodromous. Pairs of secondary veins 9–10(11), raised on underside of leaf. Tertiary veins scalariform, but scarcely visible on underside. ***Male and female inflorescences*** not seen. ***Peduncles*** 7–11 cm long, up to 0.4 cm in diam. at the base, glabrescent, grey-brown and densely lenticellate. ***Infructescence*** a woody spike, terminal, ranging from 9–10 cm. Fruits sessile on woody peduncle, spread out on spike and not clumped. Very few fruits make it to maturity, with typically 1–3 units fully ripening. ***Acorn*** globose or ovoid when developing, globose when mature, 3.3–3.8 × 2.7–3.3 cm, covered with 4–5 eccentric ridges with blunt thick coral-like spines, originating from the style scar looping towards the suture that runs along the spine of the fruit, surface puberulous. Cupule fully enclosing the nut, mostly indehiscent, but occasionally dehiscent, exposing exocarp area. Fresh cupule wall bright green, suture dark grey-black. Old cupule grey-green, darker on the surface lighter on the spines. ***Nut*** 1 in each cupule, oblong in shape, flat at the bottom, 2.5–2.9 × 2.3–2.4 cm, tip pointing down. Up to 90% of the surface area of the nut comprising scar area (receptacle tissue) and up to 10% of the surface area of the nut is slightly raised and made up of vestigial exocarp layer. Nut scar pale brown-whitish with glabrous, rugose surface, adnate to the cupule, exocarp layer light brown, covered in thin layer of silvery tomentum.

#### Phenology.

Flowering and fruiting occur annually, with flowers appearing in March and fruits maturing in July to August. Sporadic fruiting was observed in November and December 2021.

#### Distribution, habitat and ecology.

**Figure 3.**
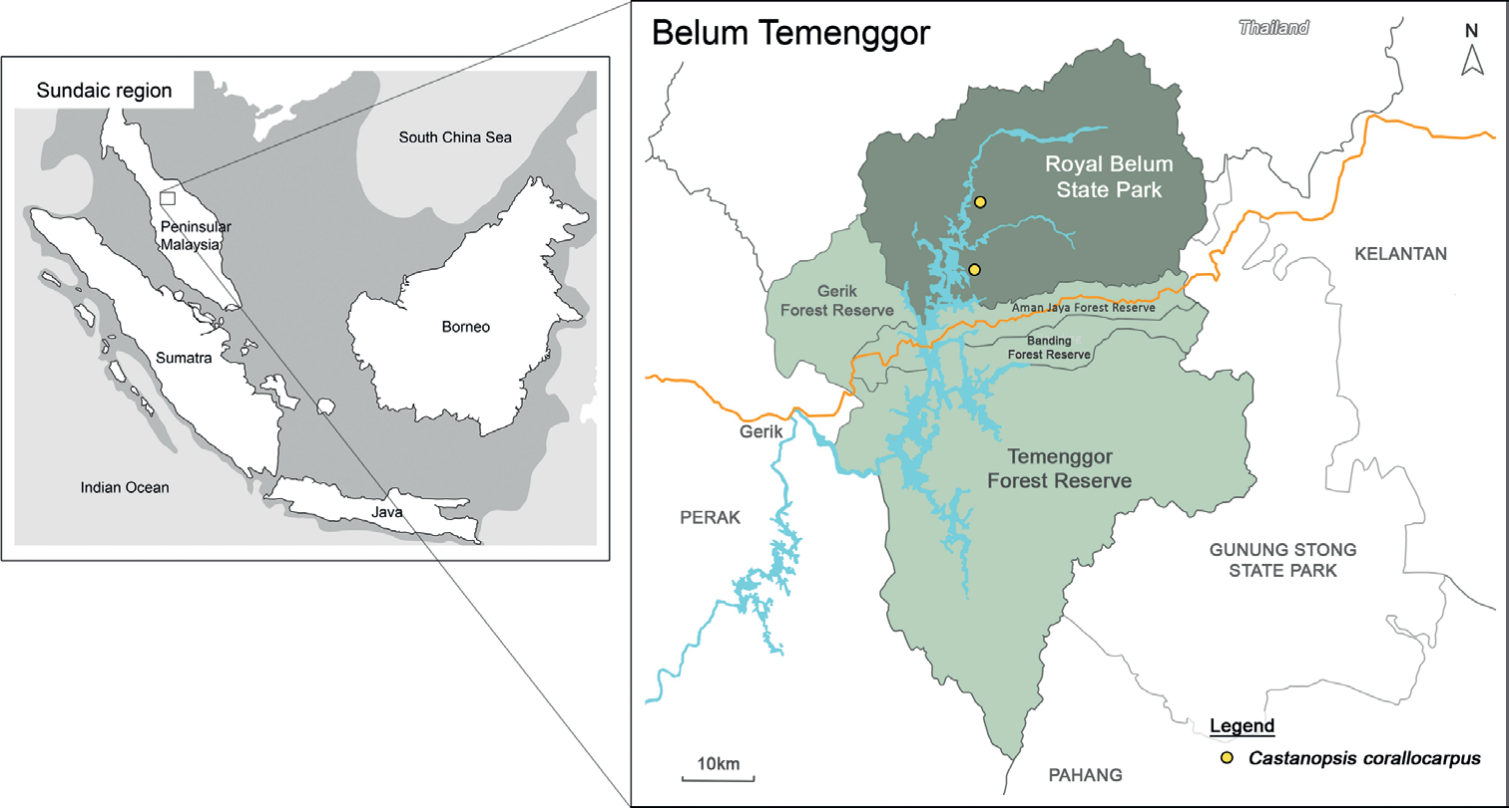
Collections sites of *Castanopsiscorallocarpus* W.H.Tan & Strijk in Belum-Temenggor Forest Complex.

*Castanopsiscorallocarpus* is only known from the Belum-Temenggor Forest Complex, Hulu Perak District, Perak, Malaysia. Within BTFC, this species has been officially recorded in Sungai Tiang and Sungai Papan as shown in Fig. 3. This species grows in both lowland and hillside dipterocarp forests (300–450 m above sea level) with a soil type of low nutrient and high clay abundance typical of most dipterocarp forest. As Peninsular Malaysia is situated near the Equator, the climate is classified as wet equatorial, characterised by high daytime temperature and high rainfall throughout the year. According to The Malaysian Meteorological Department (2022), the District of Hulu Perak experiences an average rainfall of 1500–2000 mm annually and the daytime temperature is around 27–30 °C and 21–24 °C at night with very minimal fluctuations seasonally.

#### Vernacular name.

Both indigenous communities of BTFC (i.e. the Jahai and Temiar) do not have a specific name in their language, instead referring to it in Malay as *Berangan*.

#### Etymology.

The epithet *corallocarpus*, a noun in apposition, alludes to thick coral-like spikes arranged in undulating thickened ridges on cupule of fruit.

#### Conservation status.

Based on the guidelines established by the IUCN Red List (IUCN 2022), we provide an initial assessment of the species here as Critically Endangered (CR B1B2ab(i,ii,iii,iv)), based on only two recorded individuals within Royal Belum State Park, small known range (BTFC) and the extensive habitat alteration and forest clearance throughout the immediately adjacent wider region. Although Royal Belum State Park is fully protected from logging, the southern part of BTFC namely Temenggor Forest Reserve is open to exploitation, further threatening to shrink its already small range.

## ﻿Discussion

We report the first description of a new species, *C.corallocarpus* from BTFC, Perak, Malaysia. Peninsular Malaysia has a total of 20 species of *Castanopsis* and *C.corallocarpus* is easily distinguished by the unique rows of basally reinforced, blunt coral-like spines on the cupule. At least 10 species of *Castanopsis* species have a single nut and out of these, only two have a non-spiny cupule (i.e. *Castanopsiscurtisii* King and *Castanopsisnephelioides* King ex Hook.f.). All others have either short-dense spines (*Castanopsismegacarpa* Gamble; *Castanopsismalaccensis* Gamble; *Castanopsisjohorensis* Soepadmo; *Castanopsisjavanica* (Blume) A.DC.; *Castanopsistungurrut* (Blume) A.DC.; *Castanopsisridleyi* Gamble) or thick thorns (*Castanopsisrhamnifolia* (Miq.) A.DC. and *Castanopsisacuminatissima* (Blume) A.DC.) as their main defence against seed predation.

Peninsular Malaysia and Borneo together have an estimated 103 species of Fagaceae in four genera (*Castanopsis*: 22; *Lithocarpus*: 62; *Quercus*: 18; *Trigonobalanus*: 1; Royal Botanic Gardens Kew (2021)). Numbers for the individual Bornean regions (Sabah, Sarawak, Brunei and Indonesian Kalimantan) fluctuate, depending on source and species-data considered, but it is estimated that, for the two major areas (PM and Borneo *s.l.*), endemism in Peninsular Malaysia is highest for *Castanopsis* only, whereas on Borneo, all three main genera have significant levels of endemicity (Peninsular Malaysia: C: 22%; L: 1%; Q: 0 (no endemics); Borneo *s.l.*: C: 46%; L: 91%; Q: 100% (all species endemic)). Many species in *Lithocarpus* and *Quercus* have ranges restricted to mid- or higher elevation habitat (> 600 m), which is more prevalent in Borneo (Soepadmo 1972), while occurrence and diversity of *Castanopsis* species seem to be less extremely governed by elevation, but more by the prevalence of ridges, crests, forest- and river-margins (Cockburn 1972; Soepadmo 1972).

Of the five *Castanopsis* species endemic to Peninsular Malaysia, four are found mostly in mid- to upland habitats, all first described from Perak (Larut and surrounding areas). A fifth endemic (*C.selangorensis* A.Camus) was primarily found in the lowlands, but is presumed extinct (Cockburn 1972). *Castanopsiscorallocarpus*, a sixth endemic species of *Castanopsis* for Peninsular Malaysia, differs distinctly in its distribution by occurring only in the north in the lowland and hillside forest (< 450 m). Within Malaysia and the wider region, *C.corallocarpus* is unique in its combination of properties and we further outline some of the defining differences with other species in Table 1.

**Table 1.** Morphological differences between *C.corallocarpus* and other species of *Castanopsis* of the Peninsular region.

Characters	*C.corallocarpus* W.H.Tan & Strijk	*C.inermis* (Lindl.) Benth. & Hook.f.	*C.purpurea* Barnett	*C.pierrei* Hance	*C.rhamnifolia* Miq. A.DC.
1. Cupule surface	Cupule with basally reinforced, blunt coral-like spines arranged in continuous ridges. Ridges in young fruits often terminate only a few mm apart, resulting in hollow enclosures protecting the exterior inner cupule.	Cupule sparsely covered with simple, short, curled thorns set in 3–5 lines.	Cupule sparsely covered with woody, branched, curved spines.	Cupule sparsely covered with woody, branched, curved spines.	Cupule covered with tufted, erect, simple thorn-like structures.
2. Number of cupule valves	3(–4)	2(–4)	“dehiscent”, #valves unreported	“dehiscent”, #valves unreported	3– (4?), or indehiscent
3. Number, size of acorns (*l* × *w*)	1, 3.3–3.8 cm × 2.7–3.3 cm.	(1)2–3, 1.5–2.5 cm × 1.5–3.5 cm.	1–3 (4), 4.5–6 cm in diam.	1–3 (4), 4–6 cm in diam.	1, 1–1.5 cm × 1–1.5 cm.
4. Acorn shape (ripe), position	Acorn globose, sessile and solitary.	Irregularly globose, sessile and clustered (sometimes fused).	Globose, stalked and solitary.	Irregularly globose or bilobed, semi-stalked to sessile and solitary.	Asymmetrically depressed subglobose, sessile solitary or in pairs.
5. Acorn surface	Pustulated	Pustulated	Unreported	Pustulated	Unreported
6. Nut wall	Free	Fused	Free	Free	Fused
7. Nut indumentum	Finely adpressed silver hairs on the umbo.	Finely adpressed silver hairs on the umbo.	Sparsely covered with adpressed white hairs.	Dense white hairs, only around umbo.	Unreported.
8. Nut shape, size and scar (*l* × *w*)	Rectangular and flattened at the base, 2.5–2.9 cm × 2.3–2.4 cm, scar 2.3–2.5 × 2.3–2.4 cm.	Ovoid, emarginate, concave at the bottom, 1 cm × 1 cm, scar 0.2–0.3 × 1 cm.	Ovoid and asymmetrically compressed, 2–3 cm × 1 cm, scar unreported.	Ovoid, asymmetric and slightly curved, 3 × 1.5 cm, scar unreported.	Asymmetrically depressed subglobose, 3.5–4 cm × 2–3 cm, scar basal 1 cm diam.
9. Leaf shape, size (*l* × *w*)	Elliptic, with cuneate base and acute apex, (11)13–17(19) × (2)3.5–5(6) cm.	Obovate, obovate-oblong, oblong or lanceolate, 7–17 × 5–7 cm.	Elliptic, oblong or obovate, 10–25 × 3.2–6.3 cm.	Lanceolate or oblong, 10–20 × 3–6 cm.	Oblong, elliptic or obovate, 10–20 × 3.5–8 cm.

During our field survey, we encountered additional Fagaceae species, for example, *Lithocarpuselegans* (Blume) Hatus. ex Soepadmo, *Lithocarpuswrayi* (King) A.Camus, Lithocarpuscf.kingianus (Gamble) A.Camus, *Lithocarpushendersonianus* A.Camus., *Lithocarpusmacphailii* (M.R.Hend.) Barnett, *Lithocarpussundaicus* (Blume) Rehder, *Castanopsisinermis* (Lindl.) Benth. & Hook.f., *Castanopsismalaccensis* and two (as of yet) unidentified species of *Quercus*.

Few studies have been done on Fagaceae in Peninsular Malaysia in the last decade. The discovery of *C.corallocarpus* in Peninsular Malaysia highlights the continued potential of new findings for this family in the region. Moreover, with only two recorded individuals of *C.corallocarpus* in BTFC, further assessment is needed to determine the extent of the species distribution for its conservation. This also extends to other Fagaceae species from *Castanopsis* and *Lithocarpus* in which many are data-defficient on the IUCN Red List (IUCN 2022).

## Supplementary Material

XML Treatment for Castanopsis corallocarpus
